# Case Report: Anti-Yo antibody mediated paraneoplastic cerebellar degeneration in a patient with squamous cell lung carcinoma

**DOI:** 10.3389/fimmu.2025.1558867

**Published:** 2025-03-21

**Authors:** Chen-Hao Yang, Yan Xu, Si-Yuan Fan

**Affiliations:** ^1^ Department of Internal Medicine, Peking Union Medical College Hospital, Chinese Academy of Medical Sciences and Peking Union Medical College, Beijing, China; ^2^ Department of Respiratory and Critical Care Medicine, Peking Union Medical College Hospital, Chinese Academy of Medical Sciences and Peking Union Medical College, Beijing, China; ^3^ Department of Neurology, Peking Union Medical College Hospital, Chinese Academy of Medical Sciences and Peking Union Medical College, Beijing, China

**Keywords:** paraneoplastic cerebellar degeneration, anti-Yo antibody, squamous cell lung carcinoma, case report, immunotherapy

## Abstract

Paraneoplastic cerebellar degeneration (PCD) is a heterogeneous group of neurologic syndromes associated with primary tumors. It is postulated that the immune system targets a tumor antigen that is also expressed endogenously in the nervous system. The majority of these patients are diagnosed with breast cancer or gynecological cancer, while it is exceedingly rare in lung squamous cell carcinoma (LUSC) patients. Here we reported a rare case of anti-Yo antibody-positive PCD in a patient with LUSC and got successfully treated via immunotherapy and oncological treatment. The patient’s ataxia symptoms alleviated following the administered treatments, suggesting that early immunotherapeutic intervention may have potential value in mitigating neurological deterioration. Furthermore, active and timely management of the primary carcinoma is crucial.

## 1 Introduction

PCD is a severe paraneoplastic syndrome (PNS) marked by subacute cerebellar ataxia, dysarthria, and ocular dysmetria, caused by tumor-induced autoimmunity against cerebellar antigens (1). Specific anti-neuronal antibodies in serum and cerebrospinal fluid (CSF), including anti-Yo antibodies targeting human cerebellar degeneration-related protein 2 (PCA-1), are crucial diagnostic biomarkers for PCD. Anti-Yo mediated PCD mainly affects women around 60 and is chiefly linked to breast and gynecologic cancers, including ovarian and uterine malignancies (2). While PCD is notably difficult to treat, anti-Yo PCD exhibits some of the poorest response rates to standard therapeutic interventions (3).

In this report, we present the case of a patient with PCD mediated by anti-Yo antibodies, and associated squamous cell lung carcinoma. To date, there are no more than five reported cases of PCD secondary to non-small cell lung cancer (NSCLC). (**Table 1**).

**Table 1:** The literature review of PCD patients secondary to NSCLC.

Demo	Year	Primary tumor	Antibody	Immunotherapy	Anti-tumor therapy	Prognosis	DOI
75/F	2015	LCNEC, LUAD	Anti-Ri	IVIG	Lobectomy, lymphadenectomy	Nearly complete clinical recovery	10.1016/j.jocn.2014.06.103
65/F	2020	NSCLC	anti-P/Q-type VGCC, anti-Yo	Corticoids IVIG	Pemetrexed, cisplatin	Remain ataxia, walk unassisted	10.1007/s10072-019-04139-0
67/M	2020	NSCLC	Anti-Tr3	Corticoids, IVIG	Carboplatin, paclitaxel	NA	10.1007/s00115-019-00859-y
57/M	2021	LUAD	Anti-Zic4	IVIG	Chemotherapy	Death of fatal pneumonia	10.21037/tlcr-21-989
63/M	2024	NSCLC	None	Corticoids, IVIG	NA	Death	10.7759/cureus.60258

Demo, demographics; LCNEC, large cell neuroendocrine lung carcinoma; LUAD, lung adenocarcinoma; VGCC, voltage-gated calcium channel; NA, not available.

## 2 Manuscript formatting

### 2.1 Case description

A 62-year-old female with no significant medical history presented with intermittent dry cough and left temporal headaches since August, 2023. In early September, she presented with subacute vertigo, nausea, and vomiting. The manifestations of ataxia rapidly evolved in the following week and blurred vision occurred on 15^th^, September. She gradually developed nystagmus, diplopia, ataxia of the head and perioral region. She was admitted to a local hospital. Physical examination primarily indicated coarse horizontal nystagmus in both eyes while intact strength and sensation. Benign paroxysmal positional vertigo (BPPV) test was negative. Vestibular function tests showed abnormalities in the optomotor center.

### 2.2 Diagnostic assessment

**Figure 1:**
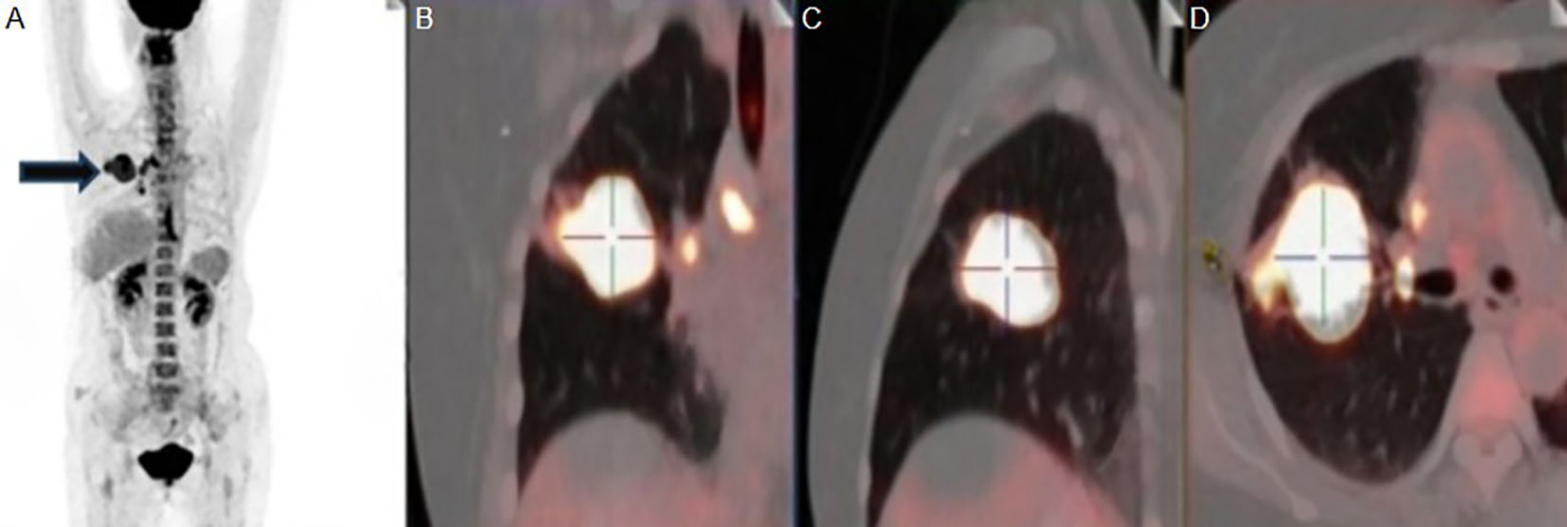
**(A)** MIP image in Positron emission tomography-computed tomography (PET-CT) of the patient and the black arrow points to the right lung lesion. **(B, C)** A solid mass in the upper lobe of the right lung with abnormal metabolism. **(D)** The apical and posterior segments of the right upper lobe are truncated by the tumor. MIP, Maximum Intensity Projection.

Cranial magnetic resonance imaging (MRI), without contrast, showed no obvious abnormalities. CSF routine and biochemistry studies returned unremarkable results. Paraneoplastic antibody profiles and anti-neuronal surface antigen antibody spectrum in blood and CSF were both negative. Symptomatic therapy was ineffective. On 30^th^, September, she was admitted to our hospital. Physical examination showed that a positive Romberg sign and gait ataxia besides original signs. Computed tomography (CT) of chest revealed that a solid mass shadow in the upper lobe of the right lung, and multiple enlarged lymph nodes were found in both hilum and mediastinum. She was considered suspected diagnosis of PCD, and then was detected abnormal in multiple serum tumor markers: Squamous Cell Carcinoma Antigen (SCCAg) 8.2ng/ml(≤2.7ng/ml), Neuron Specific Enolase (NSE) 18.1ng/ml(≤16.3ng/ml), cytokeratin 19 fragments (Cyfra21-1) 16.5ng/ml(≤3.5ng/ml). And furthermore, she was tested slightly positive for the anti-Yo antibody in the blood but negative for other antineuronal antibodies (EUROLINE Paraneoplastic Neurologic Syndrome 12 Antigen test kit, a sort of immunoblot). The contrast-enhanced MRI of head showed no obvious abnormality. The positron emission tomography-computed tomography (PET-CT) revealed a malignant lesion in the upper lobe of the right lung (**Figure 1A**), about 4.8×3.7cm in size, SUV (standard uptake values) max 64.3, slightly pulling the adjacent pleura, locally close to the oblique fissure and horizontal fissure (**Figures 1B–D**), with multiple lymph node metastasis in the left supraclavicular region, right hilar lung and mediastinum. Then histological and immunohistochemical results of the tumor punctures suggested squamous cell lung carcinoma with ALK-D5F3(-), CK7(-), P40(+), TTF-1(-), PD-L1(22C3)TPS=10%.

On 18^th^, October, she was initiated of oral prednisone with a starting dose of 30mg qd(about 0.6mg/kg/d). On 22^nd^, October, with such definitive diagnosis of squamous cell lung carcinoma related PCD, intravenous immunoglobulin (IVIG) 0.4 g/kg/d for 5 days were initiated, and subsequently her vertigo, nausea and vomiting were obviously alleviated. She eventually began to resume oral feeding, and what’s surprising to us is that she could even stumble around with support of her family. Starting on 2^nd^of November,1^st^ and 31^st^ of December, respectively, three courses of intravenous etoposide 75mg qd D1-5 and cisplatin 75mg D1, D8 were given as treatments for primary tumor. At the same time, she was given concurrent radiotherapy: 50.4Gy/28f and 60.2Gy/28f. CT showed that the tumor’s size shrank obviously. From 3^rd^ to 9^th^ in November, she had been provided with methylprednisolone sodium succinate 80mg qd, and then her tremor of head and perioral, horizontal nystagmus and gait ataxia got more lessened. Oral prednisone 60mg qd (about 1mg/kg/d), which was tapered slowly.

**Figure 2:**
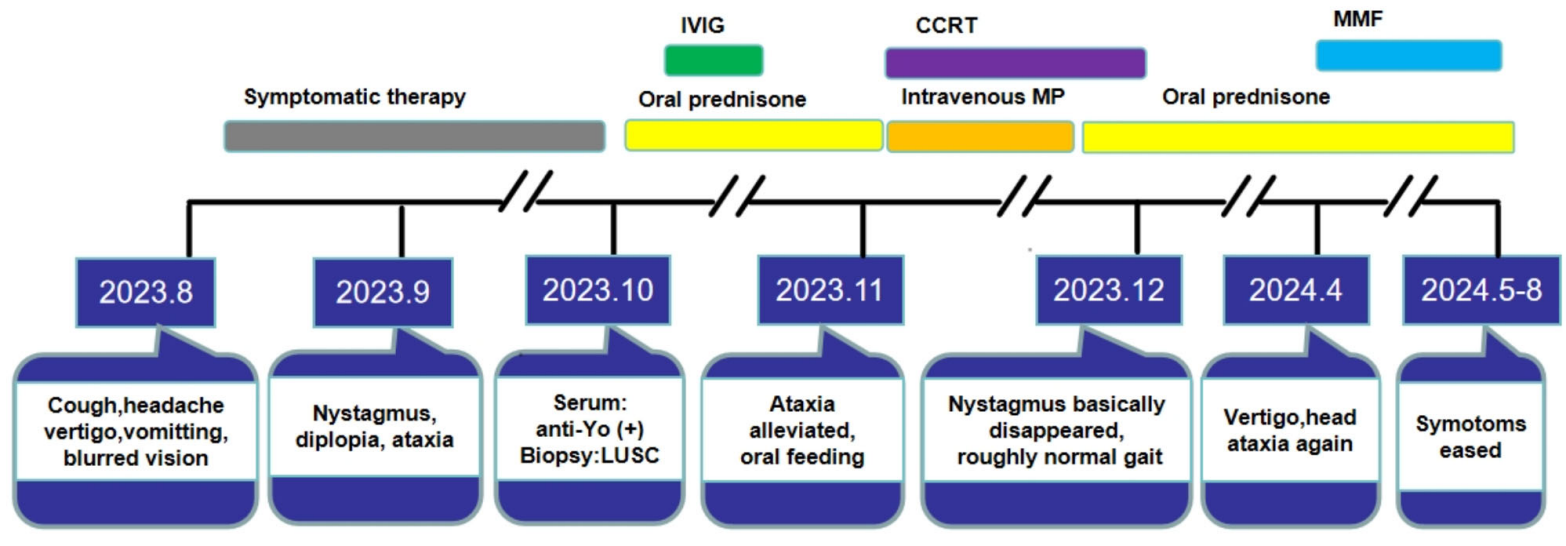
The completed timeline of diagnosis and treatment of this patient. LUSC, lung squamous cell carcinoma; IVIG, intravenous immunoglobulin; CCRT, concurrent radio chemotherapy; MP, methylprednisolone; MMF, mycophenolate mofetil.

After chemoradiotherapy and PCD-related immunotherapy, her nystagmus basically disappeared and she could walk along with roughly normal gait. Reexamination of head contrast-enhanced MRI on 25, March of 2024 was generally normal. However, when the prednisone dose was reduced to 7.5mg qd, she developed vertigo and head ataxia again. The increased dose of prednisone to 20mg qd in combination with mycophenolate mofetil (MMF) 0.5g bid was administrated to avoid recurrence. Then such symptoms got eased. The dose of MMF was gradually increased to 0.75g bid, and the prednisone dose was tapered at rate of 2.5mg every 2 weeks, where no recurrence was observed during this period. Without support, she can walk by herself and the straight-line walking test is negative. (The timeline of diagnosis and treatment is shown in **Figure 2**).

## 3 Discussion

Neurological paraneoplastic syndromes (PNS) are uncommon, affecting 1–3% of patients with cancer. Of these, 25% are associated with PCD, rendering it the most prevalent paraneoplastic syndrome affecting the brain (1).

The precise pathogenesis of PCD remains inadequately elucidated; however, it is postulated that autoimmunity mechanisms may be implicated, which hypothesize that PCD arises from a phenomenon of molecular mimicry, wherein random mutations in CDR2/CDR2L (Cerebellar Degeneration Related antigen 2/Cerebellar Degeneration Related protein 2-like) genes of cancer cells (4) may elicit T cell and antibody responses from the human immune system (5), as such antigens are expressed by Purkinje cells, ovarian, breast tissues, and other tissues. This exactly explains why PCD is more common in patients with breast or gynecological tumors. To the best of our knowledge, no more than five patients with non-small cell lung cancer have been reported to have secondary PCD. It has been documented that antibodies associated with PCD include anti-Yo, Hu, CV2, Ri, Ma, Tr/DNER, Zic4, amphiphysin, and anti-Sox1 (3). Anti-Yo targets 2 intracellular antigens, CDR2 and CDR2L, expressed in the nucleus and cytoplasm of Purkinje neurons in the cerebellum, respectively (6, 7). According to previous reports, anti-Yo antibodies may be negative in the early stages of the disease (8).The impossibility to revalidate serum anti-Yo antibodies, a key diagnostic biomarker for PCD in this patient, using CDR2L as antigen in both local and our hospitals, where actually chose CDR2 as antigen, is a limitation in our case. Because questions have been raised regarding the broad utility of CDR2 as antigen due to the potential for false results (9). Evidence suggests that by adding a test for CDR2L, which is the major Yo antigen, the sensitivity and specificity of diagnosis can be greatly improved, even to 99-100% in some reports (10–12).

Clinical manifestations of PCD are heterogeneous, primarily characterized by cerebellar dysfunction, encompassing trunk and limb ataxia, vertigo, nystagmus, diplopia, and dysarthria, which brings challenges to the diagnosis of doctors and can be easily ignored by patients. Although ataxic gait may present as the initial and most prominent manifestation, a diagnosis of rapidly progressing cerebellar syndrome necessitates the involvement of the trunk and upper extremities over a period of several months. This progression is crucial for establishing the diagnosis (13). Symptoms indicative of brainstem involvement, even without intervention, will reach their peak within 6 months (14).

The scarcity of randomized clinical trials due to the rarity of cases makes PCD a notable therapeutic challenge. Based on clinical experience, therapeutic approaches can be categorized into symptomatic therapy, acute and maintenance immunotherapy, and curative or palliative oncologic treatment. Symptomatic therapy will not be expatiated here. Regarding acute immunotherapy, corticosteroids (GCS) are typically selected as the first-line approach. From a pathophysiological perspective, corticosteroids not only target brain inflammation and edema, but also induce apoptosis of antibody-producing plasma cells (15, 16). Acute immunotherapy can also be achieved through intravenous immunoglobulins (IVIG) (17) or plasma exchange (PLEX) (18). These therapeutic modalities can augment corticosteroid treatment in severe cases or serve as an alternative intervention when corticosteroids prove ineffective. IVIG are readily administered at a dosage of 0.4 g/kg daily for 5 days and are relatively accessible. PLEX requires invasive high-volume central line placement, might induce or exacerbate hypotension, and therefore might be unfeasible for many centers. Maintenance immunotherapy aims to sustain or enhance the positive effects of the initial immunotherapy and prevent relapses. Oral corticosteroids are primarily used when there is a favorable response to methylprednisolone. Substances targeting all types of immune cells (primarily T- and B-cells) include azathioprine, MMF (19), and cyclophosphamide (20). In theory, concomitant tumor treatment is crucial in removing the source of the aberrant paraneoplastic immune response. Some reports indicate benefits from early antitumor therapies like surgery and chemotherapy (21).

The prognosis of anti-Yo-mediated PCD is unfavorable, with long-term survival rates below 25% (22). Extended studies examining patient outcomes indicate that more than 90% of individuals require ambulatory assistance, with the majority ultimately becoming bedridden (23). Two of five patients listed in **Table 1** succumbed to the condition, while the other two experienced significant disability. Our patient’s notably improved prognosis compared with previously reported NSCLC patients may be attributed to more aggressive medical intervention. IVIG and half-dose GCS were administered when PCD was strongly suspected. Upon diagnosis, concurrent chemoradiotherapy with full dose GCS was initiated. This favorable outcome may also be associated with the patient’s responsiveness to the comprehensive therapy for both PCD and LUSC.

For monitoring of efficacy, no clear correlation exists between antibody titers and the severity of neurologic symptoms. Therefore, we didn’t routinely monitor antibody levels during the patient’s treatment. For instance, antibody titers might decrease during PLEX, but symptoms may not improve (22). Additionally, the persistence of antibodies after tumor resection is common (14).

## 4 Conclusion

In summary, we reported the successful diagnoses and treatment of a 62/F patient with anti-Yo mediated PCD secondary to LUSC. For patients with highly suspected anti-Yo PCD, negative results of antibody do not necessarily indicate the absence of autoimmunity, and more aggressive medical intervention such as GCS and IVIG might be warranted. Antitumor therapy is still the cornerstone of the regimen. Different PCD patients have different responses to treatments such as GCS, and the underlying mechanisms need to be further elucidated.

## Data Availability

The original contributions presented in the study are included in the article/**Supplementary Material**. Further inquiries can be directed to the corresponding author.
