# Supplementary material for: Case Report: Anti-Yo antibody mediated paraneoplastic cerebellar degeneration in a patient with squamous cell lung carcinoma
Source: Front Immunol. 2025 Mar 21;16:1558867. doi: 10.3389/fimmu.2025.1558867 (PMC11968391; doi:10.3389/fimmu.2025.1558867)
Supplement: Supplementary file 1 [file DataSheet1.pdf]

CARE Checklist of information to include when writing a case report

CARE case report guidelines

(cc) BY-NC-ND

Topic	Item	Checklist item description	Reported on Line
Title	1	The diagnosis or intervention of primary focus followed by the words "case report"	1-2
Key Words	2	2 to 5 key words that identify diagnoses or interventions in this case report, including "case report"	13-14
Abstract (no references)	3a	Introduction: What is unique about this case and what does it add to the scientific literature?	16-20
	3b	Main symptoms and/or important clinical findings	20-21
	3c	The main diagnoses, therapeutic interventions, and outcomes	20-22
	3d	Conclusion—What is the main "take-away" lesson(s) from this case?	22-24
Introduction	4	One or two paragraphs summarizing why this case is unique (**may include references**)	25-36
Patient Information	5a	De-identified patient specific information.	39-40
	5b	Primary concerns and symptoms of the patient.	39-43
	5c	Medical, family, and psycho-social history including relevant genetic information	39
	5d	Relevant past interventions withoutcomes	49
Clinical Findings	6	Describe significant physical examination (PE) and important clinical findings.	43-46
Timeline	7	Historical and current information from this episode of care organized as a timeline	40-43,51
Diagnostic Assessment	8a	Diagnostic testing (such as PE, laboratory testing, imaging, surveys).	48-66
	8b	Diagnostic challenges (such as access to testing, financial, or cultural)	48-50,57-59
	8c	Diagnosis (including other diagnoses considered)	64-66
	8d	Prognosis (such as staging in oncology) where applicable	62-64
Therapeutic Intervention	9a	Types of therapeutic intervention (such as pharmacologic, surgical, preventive, self-care)	67-69,72-77,82-83
	9b	Administration of therapeutic intervention (such as dosage, strength, duration)	67-69,72-78,82-85
	9c	Changes in therapeutic intervention (with rationale)	75-77,82-85
Follow-up and Outcomes	10a	Clinician and patient-assessed outcomes (if available)	70-72,74-77,79-82,84-87
	10b	Important follow-up diagnostic and other test results	74-75,80-81
	10c	Intervention adherence and tolerability (How was this assessed?)	None
	10d	Adverse and unanticipated events	None
Discussion	11a	A scientific discussion of the strengths AND limitations associated with this case report	103-107,132-137,142-143
	11b	Discussion of the relevant medical literature **with references**.	90-103,114-116,121-124
	11c	The scientific rationale for any conclusions (including assessment of possible causes)	126-128,131-138
	11d	The primary "take-away" lessons of this case report (without references) in a one paragraph conclusion	142-145
Patient Perspective	12	The patient should share their perspective in one to two paragraphs on the treatment(s) they received	146-150
Informed Consent	13	Did the patient give informed consent? Please provide if requested	Yes ☑ No ☐
